# Endoprosthetic Reconstruction of distal Humerus following Resection of distal Humeral Giant Cell Tumours in Six Patients in Rural India

**DOI:** 10.5704/MOJ.1707.001

**Published:** 2017-07

**Authors:** N Balasubramanian, R Gnanasundaram, S Prakasam

**Affiliations:** Department of Orthopaedics, Saveetha Medical College & University, Chennai, India

**Keywords:** giant cell tumour, endoprosthetic reconstruction, DASH scoring, custom mega prosthesis

## Abstract

Giant cell tumour is a commonly occurring benign bone tumour in the Indian population. The common sites of involvement in descending order of frequency are distal femur, proximal tibia, distal radius and proximal humerus. The less commonly occurring sites are distal humerus, pelvis and proximal femur. We present six cases of giant cell tumour involving the distal humerus in rural India. After obtaining a tissue diagnosis by Trucut biopsy and classifying using Enneking's classification, we proceeded to perform wide resection followed by endoprosthetic reconstruction using custom mega prosthesis. We present here six patients (M: F: 2: 4) who were managed by us between 2008-2014. They presented to us with pain around the elbow and restriction in range of movements. They were each noted radiographically to have a lytic lesion involving the distal humerus with the likely diagnosis of giant cell tumour. Closed biopsy was done in all of them to obtain a definitive diagnosis. All patients underwent wide resection and reconstruction using distal humerus custom prosthesis. All patients were followed up at 6, 12, 18 and 24 weeks and thereafter six monthly until the last review. They were assessed using the DASH scoring system. All patients were well with no evidence of recurrence with good to fair functional outcome. We conclude that careful pre-operative planning with meticulous soft tissue dissection and good implant metallurgy and design, these tumours can be treated with good long term functional results.

## Introduction

Giant cell tumour (GCT) is a commonly occurring benign bone tumour occurring in the epiphyseal region of long bones. It occurs in the age group of 20-35 years in skeletally mature individuals with a slightly higher female incidence. The commonly involved sites are distal femur, proximal tibia, distal radius and proximal humerus in decreasing frequency of occurrence. However, it can occur in unusual areas like the distal humerus which is rarely reported in the literature^1–3^. In the Indian population, the occurrence of GCT is common though many cases are still unreported, but the recent trend suggests that better patient awareness and education especially in rural areas has resulted in more patients reaching hospitals for treatment of these complex problems. The treatment options are tumour resection and arthrodesis, tumour resection and reconstruction using either an allograft or a custom prosthesis. In the more extensive tumours with soft tissue involvement the safer option would have been an above-elbow amputation. Limb salvage has now replaced limb ablation as the preferred modality in selected patients. The poor availability of allografts along with the high rejection and infection rates has resulted in allografts losing favour to the more advanced custom prosthesis.

The modular custom prostheses used worldwide are generally very expensive [e.g Link™]. Here we present our series of six patients with distal humeral GCT treated in our centre. All our patients were from rural background belonging to the low socio-economic strata. Due to financial constraints, the use of more expensive custom modular prosthesis was not feasible in our study. We believe that, this is the largest series of its kind reported in literature (six cases).

## Materials and Methods

**Fig. 1:**
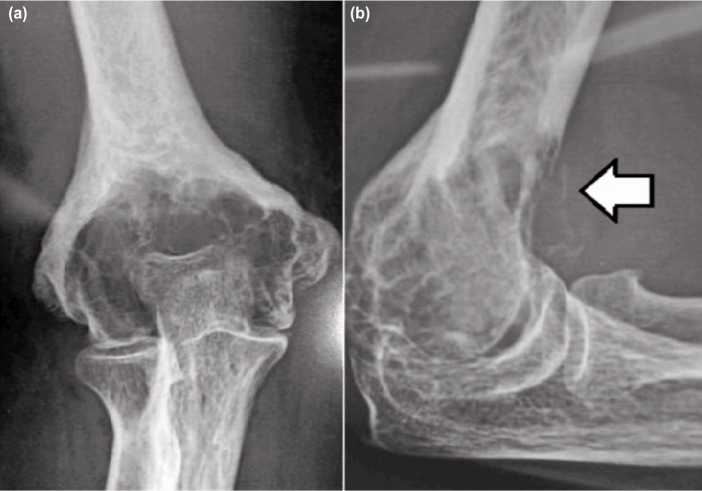
(a) Pre-operative plain radiograph of the elbow-AP showing distal humeral lytic lesion and (b) Lateral radiograph showing breach in anterior cortex (arrow).

For all the six patients, we confirmed the diagnosis with tissue biopsy and classify the tumour based on the Enneking’s classification system (four patients with stage I and two patients with stage II) (Fig. 1a, 1b). Once the diagnosis was confirmed, we proceeded to do a wide resection with endoprosthetic reconstruction using distal humeral custom mega prosthesis. Since all our patients belonged to lower socio-economic strata we used a locally made prosthesis which costs approximately 300 USD when compared to the modular systems that were available commercially (USD 1300 approx.)

**Table I:** Table showing demographic data and patient outcomes

**S No.**	**Age/Sex**	**Diagnosis/Classification (Enneking’s)**	**Follow-up (months)**	**Average DASH score**	**Outcome**	**Remarks**
1.	37/F	II	26	73	Fair	Ulnar neuropraxia
2.	31/F	II	33	76	Good	-
3.	23/M	I	28	77	Good	-
4.	34/F	I	27	71	Fair	Ulnar neuropraxia
5.	36/M	II	24	78	Good	-
6.	45/F	II	18	78	Good	-

The distal humeral custom prosthesis that was used in our study was made of 316 L surgical grade stainless steel. The dimensions of the prosthesis were calculated using the standard non-digital radiograph with specifications related to the length of the probable resection of the distal humerus, intramedullary diameter of the proximal humeral shaft and proximal ulna to accommodate the intramedullary portion of the implant both proximally and distally. The diameter of the intramedullary portion was deliberately undersized by two millimeters from the actual measurements to allow for the cement fixation. The implant was of the constrained type since both ulna and radial collateral ligaments were sacrificed during resection. This provided the medial-lateral stability for the elbow. Since the prosthesis was custom made, it took around five to seven days to manufacture based on the technical specifications offered to the implant manufacturer.

**Fig. 2:**
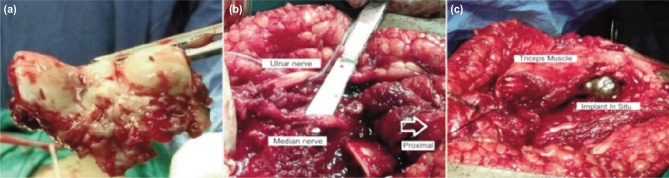
Clinical picture showing (a) resected distal humeral specimen, (b) tumour bed with isolated median and ulnar nerves and (c) implant in-situ with triceps muscle sutured over prosthesis.

All our patients were operated by a single surgeon using a standard posterior midline incision. The ulnar nerve was isolated, mobilized (Fig. 2a, 2b) and protected away from the field of dissection. Using a triceps tongue flip approach (Fig. 2c), a V-shaped flap of the triceps was mobilized distally with the olecranon to obtain exposure of the distal humerus. The length of the resected distal humerus was calculated preoperatively. The humeral shaft was prepared using 6,7 intramedullary humeral reamers. The ulnar shaft was prepared using Rush pin reamers. The proximal and distal ends of the stems were inserted into the humerus and ulna respectively and were securely fixed with bone cement. The soft tissues were carefully sutured back using 2-0 prolene, suction drain inserted and skin closed with staples.

**Fig. 3:**
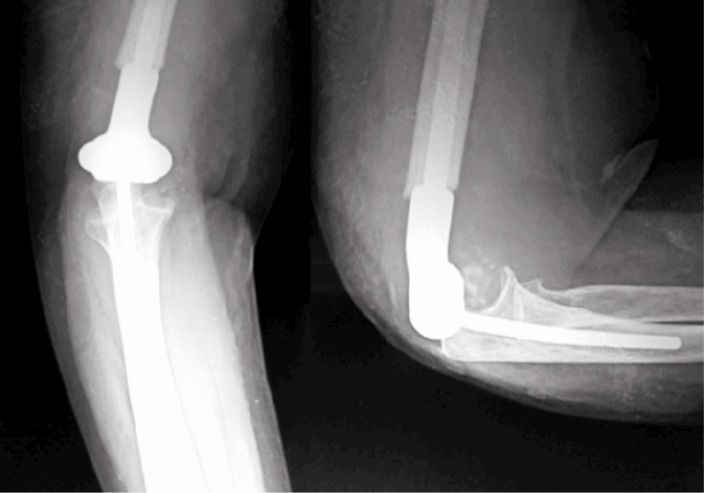
Post-operative plain radiograph showing good position of prosthesis and no evidence of recurrence or loosening at 6 months.

A posterior support above elbow slab was applied for a period of four weeks post-operatively to allow soft tissue healing. Gradual elbow movements were started after four weeks. The patients were taught to increase flexion by 5-10 degrees a day. All patients were put on Cap. Indomethacin 25 mg thrice a day for four weeks to prevent heterotrophic ossification. Serial radiographs were taken at 6,12,18 and 24 weeks, thereafter at six monthly intervals to assess tumour recurrence and loosening of the implant (Fig. 3).

## Results

**Fig. 4:**
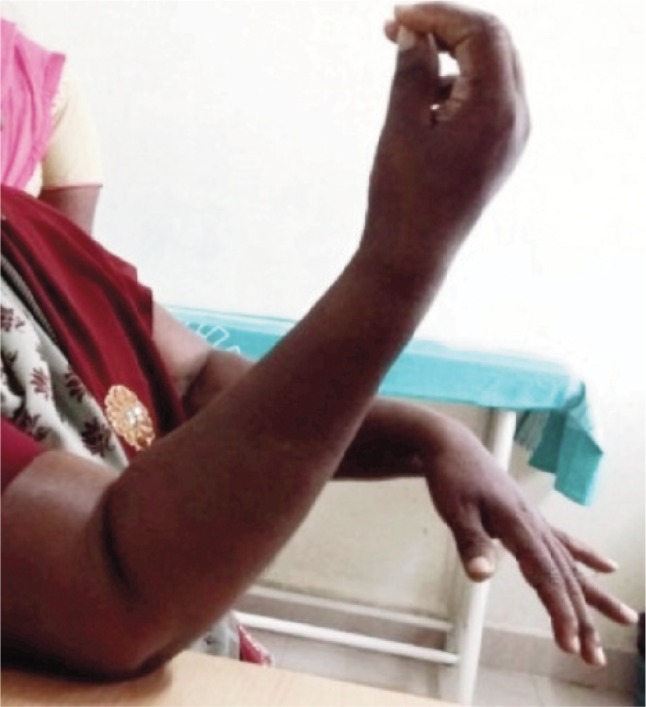
Clinical picture showing good functional outcome with 800 elbow flexion at 6 months follow-up.

There were two male and four female patients with GCT of the distal humerus. The average age was 34.33 years. Two patients developed iatrogenic ulnar neuropraxia which spontaneously recovered at ten weeks. At the end of one year follow–up of all our patients, there was no evidence of recurrence of tumour with an average quick DASH score of 77%. There was no case of implant loosening. The average range of motion achieved in our study was 92 degrees (70-105 degrees) (Fig. 4). All six patients had a fixed flexion deformity of 10-20 degrees which did not significantly affect their routine activities.

## Discussion

Hedge *et al*^1^ reported a single case of GCT in the distal humerus of a 3-year old male child who underwent a distal humerus resection and reconstruction with a sloppy hinge total elbow prosthesis. They reported at 18 month follow-up no tumor recurrence, with flexion from 15-120 degrees which is comparable to the results of our study. Liu *et al*^2^reported a series of malignant and locally aggressive GCT of the upper extremities and head and neck region. In their follow-ups they reported no local recurrence at 42 months. Our study is perhaps the largest of isolated GCT occurring exclusively in the distal humerus.

Novais *et al*^3^ reported a case of multicentric GCT occurring in the upper extremity involving metacarpals, distal radius, phalanges and humerus. All the lesions were treated with intra-lesional curettage. They reported a recurrence of lesions of the carpals and hand bones. Although our study involved a different tumour location, we did not observe any recurrence in our patients. Wirbel *et al* ^4^ reported the first ever synchronous existence of GCT and osteosarcoma occurring in the same patient. They showed that the patient had earlier been treated for osteosarcoma in the proximal tibia but now developing multicentric GCT. Karpik^5^ in his literature described the occurrence of GCT in the Polish population and clearly laid out the principles in the diagnosis and treatment of GCT. Datta *et al*^6^ performed a series of autograft+allograft reconstruction for intra-lesional curettage of well contained tumours. They reported a cure rate of 75% with a recurrence rate of 25%. In our study, due to the limited availability of allografts plus the fact that the lesions were juxta-articular, wide resection was carried out instead of intra-lesional curettage.

Fernandez *et al*^7^ reported a single case of GCT in the distal humerus treated by wide resection and prosthetic replacement. They reported a DASH score of 80 with no recurrence. The DASH score is comparable to the average score in our study (77). Guo *et al*^8^ published a series of total elbow replacement in benign and malignant tumours around the elbow. They reported a reduction in pain score mean from 3.6 to 2.0. They also reported an increase in flexion of from 30 to 80 degrees. They had one case of implant loosening from the humeral side (5 years post-operative and ulnar side (4 years post-operative). In our study a longer follow-up planned at five years will indicate if loosening occurs. The average range of flexion obtained in our study group was better than reported by Guo *et al.*

A few authors^9–11^ reported the procedure of partial resection of distal humerus and reconstruction using osteoarticular allografts. They advocated that this was a safe procedure in eccentrically placed tumours of the distal humerus. In our study, since all our patients had a more extensive uniform involvement of the distal humerus we performed a distal humerus resection followed by prosthetic replacement.

All the patients in our study presented to us relatively early in the disease staging with benign GCT. Hence, we were able to salvage the limb with resection and endoprosthesis. Two of our patients developed ulnar neuropraxia which eventually recovered. This was probably due to traction on the nerve during surgery and this could have been avoided with judicious retraction of the nerve during surgery. The other potential risks of this procedure are infection, implant breakage and implant loosening. Our study is a mid-term study and probably longer follow-ups of the patients will shed more light on the incidence of loosening of implants. There is always a theoretical chance of periprosthetic fracture of both the humerus and ulna which the surgeon must always explain to the patient.

Distal humerus GCT is a distinct but uncommon possibility and the management of such tumours requires a good tertiary care infra-structure with experienced tumour surgeons to obtain an excellent result. A high index of suspicion with good basic fundamental knowledge of tumour management principle is the keystone to a successful outcome. Above all in addition to skeletal reconstruction, of equal importance is good soft tissue cover for the implant and elbow with preservation of elbow movements.

This study does have its share of limitations like relatively short follow-up, smaller series of patients and more importantly no long-term survival reports of these indigenous custom prosthesis. When compared with the more advanced systems of total elbow prostheses, our implant design may appear quite basic. However, we record that our mid-term reports of using these locally made custom prosthesis has provided encouraging results thusfar.

## Conclusion

Distal humerus GCT occurs rather infrequently in the Indian population. The treatment of choice for tumours not invading the cortical surface is still extended curettage and bone cement with or without bone graft. The scenario changes once the tumour has breached the cortical surface. The only appropriate line of management in such extensive un-contained lesions is resection followed by endoprosthetic reconstruction. The use of locally produced custom prosthesis in such patients has yielded encouraging results in the midterm with long term results not available yet. The results with a functional stable elbow with slight decrease in range of movements while achieving limb salvage are favourable.
